# Clinical Application of Steroid Profiles and Their Interpretation in Adrenal Disorders

**DOI:** 10.3390/diagnostics16030381

**Published:** 2026-01-24

**Authors:** Indra Ramasamy

**Affiliations:** Conquest Hospital, The Ridge, Hastings TN37 7RD, East Sussex, UK; indrar@ozemail.com.au

**Keywords:** steroid profile, mass spectrometry, adrenal incidentaloma, congenital adrenal hyperplasia, Cushings syndrome, primary aldosteronism

## Abstract

Serum and urinary steroid profiles are altered in hormone-producing adrenal adenomas, Cushing’s or Conn’s syndrome, or adrenocortical carcinoma. Definitive diagnosis of inherited congenital adrenal hyperplasia is usually accomplished by measuring the blood levels of adrenal hormones and precursor steroids. Neonatal diagnosis of congenital adrenal hyperplasia is complicated. Alternative methods such as gas chromatography/mass spectrometry or liquid chromatography/mass spectrometry have been used for the diagnosis of congenital adrenal hyperplasia. This review covers the current application of gas chromatography/mass spectrometry or liquid chromatography/mass spectrometry in the interpretation of steroid profiles in different clinical and diagnostic settings. In the future, mass spectrometry may provide more information to assist in the choice of routine DNA analysis.

## 1. Introduction

For many years, alterations in steroid profiles have been investigated for steroid related endocrine disease. Steroid hormones are synthesised in the adrenal cortex, gonads, and placenta [1]. Knowledge of steroid biosynthesis can lead to a greater understanding of associated disorders such as hypercortisolism (Cushing’s syndrome), hyperaldosteronism (primary aldosteronism and Conn’s), and their subtypes [2,3]. Patients with congenital adrenal hyperplasia can demonstrate multiple-steroid hormone imbalance as a result of changes in steroid synthesis and present different clinical and biochemical phenotypes [4]. Adrenal incidentalomas or adrenal masses detected incidentally can either be nonfunctioning adrenocortical adenomas; adrenocortical carcinoma, though rare; or hormone-producing adenoma. The European Society of Endocrinology (ESE) guidelines [5] suggest that an adrenal incidentaloma requires an endocrine work up for adrenal hormone excess: pheochromocytoma, Cushing’s syndrome, and primary aldosteronism. While the 2016 European Society of Endocrinology guidelines recommended that all patients with adrenal incidentalomas require testing to exclude pheochromocytoma, the updated 2023 version suggests testing in patients with features not typical of benign adenoma. In patients with coexisting hypokalaemia and hypertension, the guidelines recommend the use of the aldosterone/renin ratio to investigate primary aldosteronism [5]. If adrenocortical carcinoma is suspected, both sex steroids and steroid profiling are suggested. The question of how the laboratory can best contribute to differential diagnosis in the context of disordered steroidogenesis remains.

Traditionally, clinicians have used routine clinical biochemistry tests for single steroid analysis. Recently, the application of gas chromatography–mass spectrometry (GC-MS) and liquid chromatography–mass spectrometry (LC-MS/MS) for the measurement of steroids has led to multicomponent analysis of serum and urine steroids. There has been an interest in the quantification of an increasing number of steroids, multi-steroid profiling, to obtain a better understanding of endocrine pathologies and to improve diagnostic strategies. This review will evaluate the advances in steroid measurement using tandem mass spectrometry and their use in the diagnosis of clinical disease in the context of existing investigative practice. A good understanding of steroid biosynthesis is required to interpret biochemical findings. This article will summarise the knowledge on the synthesis of glucocorticoids and mineralocorticoids, prior to discussing the role of laboratory participation in the diagnosis of causes of disordered steroidogenesis.

### 1.1. Human Steroid Metabolism

Steroid hormones are made in the adrenal cortex, gonads, and placenta. Cholesterol is the precursor for the biosynthesis of steroid hormones, and plasma lipoproteins are regarded as the principal source of cholesterol used for steroid biosynthesis.

The adrenal cortex synthesises mineralocorticoids and glucocorticoids, as well as the adrenal androgen precursors and androgens. Three functional zones of the cortex are responsible for the production of (i) aldosterone (in the outer zone of the adrenals, termed the zona glomerulosa) under the control of the renin–angiotensin–aldosterone system; (ii) the primary glucocorticoid cortisol (in the middle zone, the zona fasciculata); and (iii) the androgen precursors (in the innermost zone, the zona reticularis), dehydroepiandrostenedione (DHEA), its sulphate (DHEAS), androstendedione (A4), and 11β-hydroxyandrostenedione (11OHA4). The hypothalamus produces corticotropin-releasing hormone (CRH) that stimulates the corticotrope cells to release adrenocorticotropic hormone (ACTH), which increases the production of cortisol and DHEA by the adrenal gland. Glucocorticoids receive negative feedback on this pituitary–hypothalamus axis, while there is no feedback inhibition of the axis by the adrenal androgens [6].

**Figure 1:**
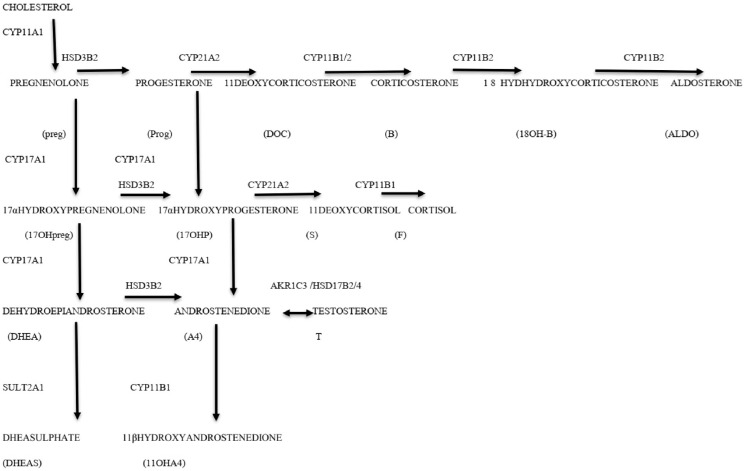
Schematic view of adrenal steroidogenesis.

The cytochrome P450 (CYP) and hydroxysteroid dehydrogenase (HSD) enzymes are the two main classes of enzymes that participate in the biosynthesis of steroid hormones. The CYPI enzymes are localised in the inner mitochondrial membrane and are reliant on ferredoxin and ferredoxin reductase for their delivery of electrons from NADPH. The CYPII enzymes are found in the endoplasmic reticulum and are dependent on Flavin adenine nucleotide and Flavin mononucleotide for electron delivery from NADPH. The HSD enzymes are divided into two families depending on their structure. The function of HSD enzymes is to catalyse the conversion of a specific steroid into its equivalent ketosteroid counterpart and vice versa (Figure 1) [6].

### 1.2. Synthesis of Specific Steroids

The increased expression of HSD3B2 in the zona glomerulosa and the lack of CYP17A1 expression ensured steroid synthesis is directed towards aldosterone biosynthesis in the zona glomerulosa. CYP11B2, also known as aldosterone synthase, converts corticosterone into aldosterone.

In humans, the zona reticularis of the adrenal cortex develops during adrenarche during ages 6–10. During that time, the decreased expression of HSD3B2 results in an increased synthesis of adrenal androgen precursor production [7]. The majority of dehydroepiandrosterone (DHEA) is converted into DHEA sulphate (DHEAS), which is an important steroid in circulation, by the DHEA sulfotransferase [8]. Low levels of HSD3B2 in the zona reticularis results in the formation of androstenedione (A4) and testosterone (T) [9].

**Figure 2:**
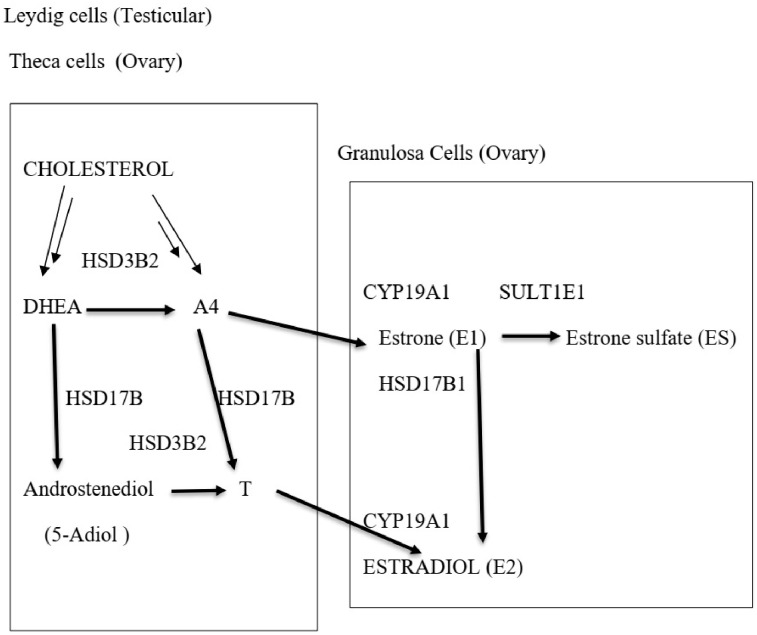
Steroidogenesis in the gonads. Several steps in steroid synthesis shown by multiple arrows.

In the Leydig cells of the testis, in the absence of SULT2A1, the major sulfotransferase, DHEA, is converted into testosterone through androstenedione and androstenediol. Testicular androgen output in the testis is mainly testosterone with lower levels of A4 and DHEA [10]. The pathway for ovarian synthesis of testosterone is similar to that of the testis. Androgen and androgen precursor production by the ovary follows a similar route to that of the testes. Ovarian steroidogenesis in the theca cells results in the production of A4 and testosterone, which enter the granulosa cells where CYP19A1 (aromatase) converts testosterone into oestrogens (Figure 2) [11].

**Figure 3:**
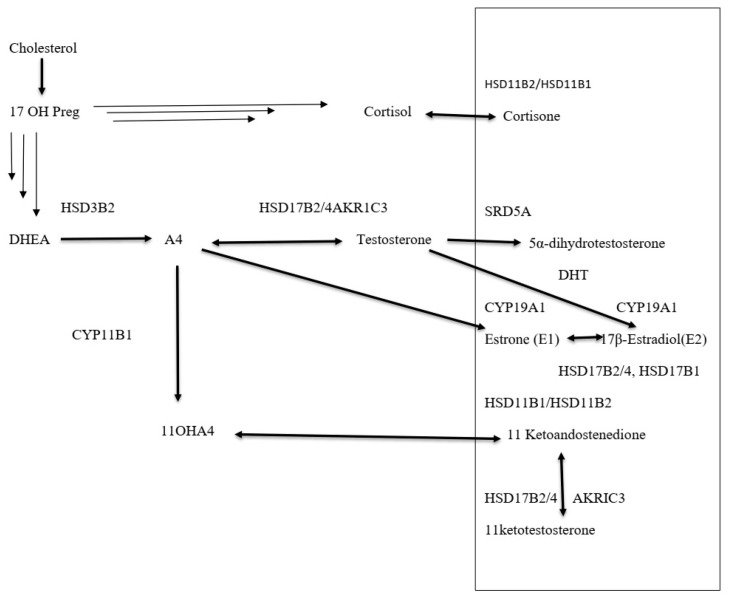
Peripheral metabolism of steroids (box indicates peripheral effects; multiple arrows show several steps).

Most of the androgen precursors formed by the adrenal and ovaries are inactive. These are converted into active androgens in target cells, which express the required enzymes. DHEA can be converted into A4 in peripheral cells, with A4 converted into testosterone by AKR1C3. Intracellular enzymes convert high circulating levels of DHEA into active sex steroids (oestrogens and androgens) in peripheral tissues ([12,13], Figure 3).

### 1.3. The Alternative DHT Pathway

**Figure 4:**
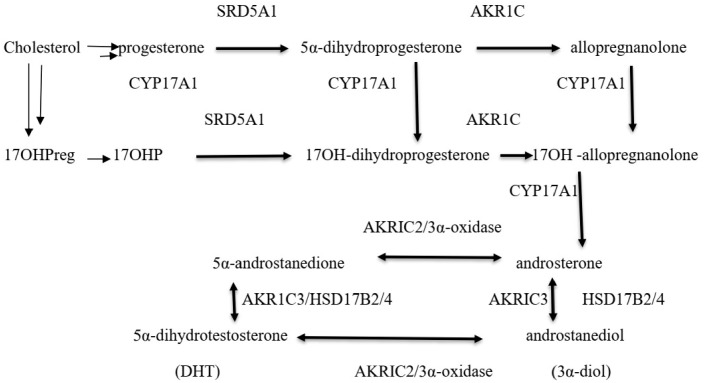
Alternative DHT biosynthesis pathway. Several steps in steroid synthesis shown by multiple arrows.

The ‘backdoor pathway’ to DHT formation bypasses A4 and T and uses androstanediol (3α-diol) as its immediate precursor to DHT (Figure 4, [14]). Data suggests that both the classical and alternative pathways are required for normal foetal masculinisation and that androgens formed by nongonadal, placental, and adrenal tissues are likely to be involved [15]. Investigations suggest that non-classic 21 hydroxylase deficiency may enhance the alternative DHT pathway [16].

### 1.4. 11-Oxygenated Androgen Pathway

**Figure 5:**
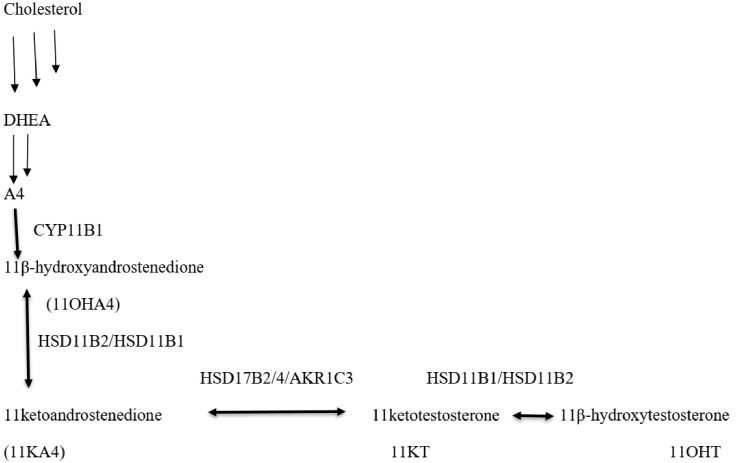
Synthesis of 11-oxygenated androgens. Several steps in steroid synthesis shown by multiple arrows.

11OHA4 is converted in peripheral tissues into 11-ketotestosterone (11KT), which is a potent androgen (Figure 5). 11 keto-5α dihydrotestosterone (11KDHT) has similar androgenic activity to DHT though its circulating levels are significantly lower than that of DHT [17]. 11-oxyandrogens have been shown to contribute to disorders of adrenal excess, such as congenital adrenal hyperplasia, premature adrenarche, polycystic ovary syndrome, as well as castration-resistant prostate cancer [18].

## 2. Methods of Steroid Analysis

In clinical laboratories, high-sensitivity immunoassays are the standard techniques for routine measurement of steroids. Large throughput is an advantage in these assays. Disadvantages are cross reactivity with structurally related compounds, which may affect steroid analysis. Other disadvantages are interference with heterophilic antibodies and high dose hook effects if sandwich assays are used [19].

Later techniques described the application of GC-MS and LC-MS/MS for the measurement of steroids. Many still regard GC-MS as the gold standard for steroid analysis; other authors consider GC-MS and LC-MS as complementary techniques. Previously immunoassays have been the prevailing analytical method, the arrival of MS techniques offer a steroid profile with a wide range of steroid concentrations [20]. Abnormalities in steroid biosynthesis and enzyme deficiencies with altered steroid biosynthesis can be found in patients with complex diseases. Steroid profiles are complex and interpretation can be a challenge. A comprehensive review dealing with technical aspects of the clinical application of steroid measurements can be found in Honour 2024 [21].

The steroid DHEAS remains the most abundant steroid in circulation (1–10 µmol/L). Serum aldosterone and its precursors DOC, corticosterone, and 18-hydroxycorticosterone (100–1000 pmol/L) are detectable in serum. Serum levels of progesterone, 17OHP, and 11-deoxycortisol are ≤10 nM; cortisol, 100–600 nM; and cortisone, 30–199 nM. Circulating steroids differ in their relative concentrations in both serum and urine [6]. Reference ranges for steroids are influenced by the specific assay methodology used. Well-defined reference ranges covering the different age ranges (including paediatric reference ranges and pubertal development) and gender are of importance in the interpretation of steroid profiles [22]. In MS methods, a stable isotope-labelled steroid as an internal standard for each endogenous labelled steroid is required but not always obtainable [21].

## 3. Cushing’s Syndrome

The differential diagnosis of Cushing’s syndrome can be complex. The diagnosis of Cushing’s syndrome is a multistep process, which includes a diagnosis of endogenous hypercortisolism, followed by determination of its aetiology. Once hypercortisolism is confirmed, the second stage is to confirm if it is ACTH-dependent. Some causes of ACTH-independent Cushing’s syndrome are unilateral adrenal adenoma, bilateral macronodular hyperplasia, micronodular hyperplasia, or adrenocortical carcinoma. Confounding factors that give rise to functional activation of the pituitary adrenal axis are psychiatric disorders, alcoholism, obesity, eating disorders, and polycystic ovary syndrome. Distinguishing pituitary-dependent ACTH secretion (Cushing’s disease) and ectopic secretion of ACTH associated with neoplasm represents a further challenge to clinical and biochemical diagnosis [23].

In the clinical laboratory, the complex panel of diagnostic tests used to diagnose cortisol secretory status are 1 mg overnight dexamethasone suppression test, urinary free cortisol in a 24 h collection, late-night salivary or plasma cortisol to show the absence of diurnal rhythm, and measurement of ACTH required for the diagnosis of Cushing’s syndrome. Diagnostic tests for the differential diagnosis of pituitary-dependent and ectopic neoplastic-associated ACTH secretion are dynamic tests with CRH stimulation. Most pituitary tumours respond to CRH, while ACTH secreting non-pituitary tumours show a rare response. Bilateral inferior petrosal sampling with or without CRH or desmopressin stimulation remains the criteria for distinguishing between ectopic and pituitary sources of ACTH [23]. All available tests for Cushing’s syndrome, however, have pitfalls [24].

Studies suggest that various causes of Cushing’s syndrome showed particular serum steroid footprints [25]. Using LC/MS/MS techniques, Eisenhofer et al. [2] showed that increases in plasma steroids, 11-deoxycortisol, 21-deoxycortisol, 11-deoxycorticosterone, corticosterone, and cortisol were found in patients with Cushing’s syndrome. Patients with adrenal disease showed the lowest concentrations of androgens, whereas those with ectopic and pituitary disease showed the lowest concentrations of aldosterone. The authors suggest that plasma steroid panel (steroid metabolomics) was both a supplementary test to screen for Cushing’s syndrome and a further guide for testing for Cushing’s syndrome aetiology. Using LC-MS/MS and analysis of plasma steroid profiles, Masjkur et al. [26] suggest that plasma 11-deoxycortisol, 11-deoxycorticosterone, DHEA, DHEA-S, and corticosterone levels can distinguish patients with and without mild autonomous cortisol secretion. In a further study that utilised high-resolution accurate mass spectrometry 24 h urine steroid profiling, etiocholanolone, 11β-hydroxyandrosterone, and α-cortolone distinguished pituitary Cushing’s disease from adrenal Cushing’s syndrome [27].

## 4. Adrenal Incidentalomas

Adrenal incidentalomas are adrenal masses discovered on imaging studies for unrelated clinical conditions. The clinical significance of adrenal incidentalomas varies based on tumour size, hormonal activity, and imaging features [5]. Biochemical testing is necessary to assess hormone excess and include steroid hormones (mineralocorticoid, glucocorticoid, and androgens) as well as catecholamines [28]. Recent studies suggest that the concurrent measurement of adrenocortical steroids and catecholamines and metanephrines in serum was possible using an LC/MS system [29]. While primary adrenocortical carcinoma (ACC) remains rare, distinguishing between hormone-secreting nodules or a nodule with an increased risk of malignancy that presents a risk to patients’ health becomes of significance. A non-contrast CT homogeneous appearance with HU ≤ 10, adrenal tumours measuring <4 cm, and rapid washout CT following contrast is more consistent with benign adrenal incidentaloma. Indeterminate nodules may require further imaging. Image-guided biopsy is not usually indicated for adrenal masses due to biopsy-associated risk [28]. ESE guidelines recommend the use of adrenal biopsy in hormonally inactive lesions (particularly, if phaeochromocytoma has been excluded); however, lesions not conclusively categorised as benign by imaging and clinical management of the patient can be altered by knowledge of histology [5].

The EURINE-ACT prospective multicentre study measured 15 urinary steroid metabolites in a 24 h sample by LC-MS/MS and application of a machine learning algorithm as a diagnostic basis for ACC. The best diagnostic performance was provided by a combination of tumour diameter greater than 4 cm, tumour HU greater than 20, and urine steroid profile indicating a high risk of ACC with a PPV of 76.4% and a negative predictive value of 99.7% [30]. Other studies suggest that urinary tetrahydro-11-deoxycortisol measured by GC/MS had a high sensitivity and specificity to differentiate between ACC and benign adrenal mass [31]. Observations suggest that urinary steroid profiles might be an additional diagnostic tool to determine if an adrenal tumour is either malignant or benign.

Recent work suggests that molecular characterisation of ACC and immune-subtyping may provide information for clinical prognostic stratification of ACC [32,33].

## 5. Primary Aldosteronism

Primary aldosteronism (PA) is caused by autonomous aldosterone secretion from the zona glomerulosa of the adrenal cortex. Screening using the aldosterone to renin ratio (increased autonomous secretion of aldosterone and consequent renin suppression) has been used as an indicator for PA. The interpretation of aldosterone to renin ratio has limitations, nearly all common antihypertensive drugs (other than α-adrenergic blockers and non-dihydropyridine calcium channel blockers, which show minimal effects [34]) interfere with the interpretation, as do nonsteroidal anti-inflammatory drugs and oestrogen-containing contraceptives. Pre-analytical errors, instability of renin at refrigerated temperatures, and hypokalaemia during sample collection can further influence results [34]. Two main types of assays exist for renin: direct renin concentration, which is based on mass measurement, and plasma renin activity, which measures renin’s ability to convert angiotensinogen into angiotensin I over time. LC-MS/MS techniques have been used to measure angiotensin I for plasma renin activity calculation, as well as blood aldosterone levels, in addition to immunoassays. However, assay-specific reference ranges for calculated aldosterone to renin ratios are required [34]. Further tests, for example, oral sodium loading, saline infusion, fludrocortisone suppression, and captopril challenge, have been used as confirmatory tests. Adrenal venous sampling is recommended in selected patients to make the distinction between unilateral and bilateral adrenal disease. Surgical treatment is the preferred option for unilateral PA (aldosterone-producing adenoma or unilateral adrenal hyperplasia), and medical treatment (mineralocorticoid receptor antagonists) is recommended [35,36] for PA with bilateral adrenal disease. Computed tomography (CT) can be used to assist the interventional surgeon and to identify large masses that may represent adrenocortical carcinoma [34].

The genes responsible for familial hyperaldosteronism (FH) types I, II, III, and IV are *CYP11B2*/*CYP11B1* *chimeric gene*, *CLCN2*, *KCNJ5*, and *CACNA1H*. FH type I was identified as a fusion of the promoter sequence of the *CYP11B1* gene with the coding region of the *CYP11B2* gene, which explains why the latter gene *CYP11B2*, coding aldosterone biosynthesis, is responsive to ACTH and to dexamethasone suppression. FH type 2 is associated with variants in the chloride channel gene *CLCN2*, which are gain-of-function mutations, enhanced chloride efflux, and increased CYP11B2 expression. In FH type 3 cases, *KCNJ5* mutations cause depolarisation of the cell with entry of Ca^2+^ ions and increased production of aldosterone. Additional genetic causes of FH are mutations in *CACNA1H* (FH type 4) and *CACNA1D* (primary aldosteronism with seizures and neurologic abnormalities) genes coding for the T-type Cav3.2 and L-type Cav1.3 voltage-gated calcium channels, respectively. It has been suggested that these mutations are aldosterone-driver somatic mutations in genes encoding ion transporters/channels/pumps that increase cytosolic calcium activity, causing increased CYP11B2 expression and aldosterone biosynthesis. As molecular and pharmacological knowledge increases, the development of specific drugs that interfere with the function of mutated channels can lead to personalised treatment for FH [3].

Studies have suggested that steroid profiling can be useful in the diagnosis of PA [37,38]. In a further study, plasma specimens were analysed by LC-MS/MS, and results analysed by machine learning algorithms. Eight steroids—aldosterone, 18-oxocortisol, 18-hydroxycortisol, 11-deoxycorticosterone, cortisol, cortisone, androstenedione, and dehydroepiandrosterone—in combination with the aldosterone and renin ratio best distinguished primary hypertension from PA. Plasma 18-oxocortisol was higher in patients with unilateral aldosterone-producing adenomas due to *KCNJ5* variants, other than other causes of PA. The presence of *KCNJ5* was independently associated with biochemical cure, and the post-adrenalectomy biochemical cure rate was 96.6% in patients with *KCNJ5* variants and 83.6% of patients without *KCNJ5* variants [39]. Prete et al. [40] used the GC-MS technique to analyse 24 h urine samples for 34 different steroid metabolites, which included mineralocorticoids, glucocorticoids, androgens, and their precursors. All 34 steroid metabolites showed an increase in the identification of PA cases vs. healthy normotensive controls (AUC-ROC = 0.97 ± 0.03); among these, the three metabolites 3α,5β-tetrahydroaldosterone, tetrahydro-11-deoxycortisol and 18-hydroxytetrahydro-11-dehydrocorticosterone were the most discriminative. Linear regression analysis showed that urine excretion of the steroid 18-hydroxycortisol and its metabolite 18-oxo-tetrahydrocortisol identified patients with somatic *KCNJ5* mutations compared to other aldosterone-producing adenomas. One hypothesis is that intratumoural steroidogenesis can be influenced by CYP11B1 and CYP11B2 enzymes. Studies suggest an inverse correlation between 18-hydroxycortisol and staining intensities for CYP11B1 enzymes. Though further studies are required to comprehend steroid metabolomics in PA [41,42]. Arlt et al. [43] used 24 h urine collections and GC-MS to recognise 32 different steroid metabolites derived from mineralocorticoids, glucocorticoids, androgens, and their precursors. They report significant excess glucocorticoid secretion in primary aldosteronism patients and an increased mineralocorticoid output in Cushing’s patients. This suggests that the differences between Cushing’s and Conn’s are not as clear as assumed earlier. This indicated the presence of Connshing syndrome or adrenal-related overproduction of aldosterone and cortisol. Steroid metabolomics may be a useful step in advancing the understanding of Connshing syndromes [44]. Cortisol secretion can make the interpretation of adrenal venous sampling, considered the gold standard in distinguishing unilateral and bilateral secretion of aldosterone, difficult [45].

## 6. Congenital Adrenal Hyperplasia (CAH)

CAH is an inherited autosomal recessive disorder that results in errors in cortisol/aldosterone synthesis. More than 90% of cases of CAH are caused by 21-hydoxylase deficiency (21OHD) as a result of mutations in the *CYP21A2* gene [46]. The phenotypic variations in the allelic variants of the *CYP21A2* gene are on a continuum based on either absent (the classic form) or reduced (non-classic form) residual enzymic activity. The severely affected form with absent or severe deficiency in the enzyme presents as a salt-wasting form in the neonate, with hyponatremia, hyperkalaemia, acidosis, and shock. There is excess androgen production as elevated steroids are transferred to the androgen synthesis pathways. Severely increased prenatal adrenal androgen production leads to virilisation of female external genitalia. Individuals with milder allelic variants and increased residual enzyme activity generally present following infancy and may have basal cortisol and aldosterone levels within the reference range, with slightly elevated androgens. Females with poorly controlled CAH may show acne, female hirsutism, male pattern baldness, altered body habitus, irregular menses, and subnormal fertility, and males may develop benign testicular adrenal rest tumours and primary or secondary gonadal failure [47].

Newborn screening for 21OHD detects salt-wasting adrenal crisis and prevents further morbidity and mortality thanks to early presymptomatic treatment. The biochemical marker for 21OHD is 17OHP, the main substrate for the enzyme found upstream of the block. There was a high false positive rate for 17OHP when using immunoassay methods [48]. LC-MS/MS was suggested as a second-line screening test for dried blood spots, and a ratio of (17OHP + androstenedione)/cortisol was used in screening tests [49]. Other analytes used in neonatal screening for 21OHD using an LC-MS/MS serum steroid profile were 11-deoxycortisol, 21-deoxycortisol, and cortisol. A metabolic block in 21OHD CAH leads to the accumulation of 17OHP, androstenedione, and 21-deoxycortisol and reductions in 11-deoxycortisol and cortisol [50,51].

Although 21-hydroxylase deficiency is the most common form of CAH, other causes are also observed. 11 β-hydroxylase deficiency (11 β-OH deficiency, CYP11B1 deficiency) is the most common type of CAH after 21-hydoxylase deficiency, with a prevalence of 1/100,000 live births. CYP11B1 converts 11 deoxycortisol into cortisol in steroidogenesis in the adrenal cortex. There is a spectrum of clinical presentation depending on the level of CYP11B1 deficiency. CYP11B1 deficiency leads to high levels of 11-deoxycortisol and 11-deoxycorticosterone, which results in increased androgen synthesis pathways and high levels of androgenic steroids. This may result in virilisation and significant masculinisation of external genitalia in female newborns. Extensive application of gene sequencing techniques are used in the diagnosis of CYP11B1 deficiency [51,52]. Feng et al. [53], using dried blood spots and a microbore LC-MS/MS technique, report that the levels of 11-deoxycorticosterone, 11-deoxycortisol, testosterone, DHEA, DHT androstenedione, and 17-OHPreg were significantly increased in a CYP11B1 deficiency group. In a single case study, urinary steroid excretion using GC-MS suggested that an increased ratio of Tetrahydro 11-deoxycortisol/(Tetrahydrocortisone + tetrahydrocortisol + 5α-tetrahydrocortisol) identified a patient with CYP11B1 deficiency [54]

The enzyme 3β-hydroxysteroid dehydrogenase type 2 deficiency (HSD3B2 deficiency) is a rare type of deficiency with <1/1,000,000 estimated prevalence at birth. This is characterised by impaired steroid synthesis in gonads and adrenal glands, which leads to decreased cortisol, aldosterone, and androstenedione concentrations and increased renin, ACTH, and DHEA, with the latter being converted into testosterone by extra-adrenal HSD3B. Elevated Δ5-17-hydroxypregnenolone has been stated as a marker of HSD3B2 deficiency. As HSD3B2 catalyses the conversion of Δ5-steroids (pregnenolone, 17-hydroxypregnenolone, DHEA, and androstenediol) to Δ4-steroids (progesterone, 17OHP, androstenedione, and testosterone), the hormonal changes observed in patients with HSD3B2 deficiency are high ratios of the Δ5- over Δ4-steroids. ACTH stimulation tests can be used for the diagnosis of HSD3B2 deficiency, and molecular testing can be used to confirm diagnosis. The clinical staging varies according to the severity of the genetic mutation and may include salt-wasting in both sexes, incomplete masculinisation in males, and virilisation in females [55]. Using plasma samples and LC-MS/MS technology Guran et al. [56] confirmed increased ∆5 steroids in the 3βHSD2 deficiency phenotype, and lower concentrations of cortisol compared to controls. The oxyandrogens did not show a difference between patient and control groups. They suggest that a high baseline 17OHPreg to cortisol ratio and lower 11-oxyandrogen concentrations by LC-MS/MS unequivocally identified patients with 3βHSD2 deficiency. In a single patient with a 3βHSD2 deficiency urinary steroid profile by GC-MS analysis revealed excessive amounts of Δ5 steroids and extremely low levels of cortisol metabolites [57].

17α hydroxylase/17–20 lyase deficiency (17OHD) is a rare autosomal recessive form of CAH caused by biallelic mutations in the *CYP17A1* gene. The gene encodes cytochrome p450c17 and has 17α-hydroxylase (pregnenolone to 17-OH pregnenelone is decreased, and progesterone is increased compared to 17-OH progesterone) and 17,20 lyase activities (17-OH pregnenolone to DHEA and 17-OH progesterone to androstenedione is decreased; concentrations were measured by radioimmunoassay). It accounts for about 1% of all CAH forms, with a prevalence rate of 1 in 50,000 newborns. Mutations in *CYP17A1* resulted in decreased cortisol and sex steroid production, resulting in sexual infantilism and pubertal failure, with increased mineralocorticoid precursors causing hypertension and hypokalaemia. 17α hydroxylase/17–20 lyase deficiency can present as complete or partial deficiency [58,59]. Sun et al. [60] utilised urinary steroid profiling by GC-MS to characterise 17α hydroxylase/17–20 lyase deficiency in eight patients. The 17α-hydroxylase activity was measured by (tetrahydro-11-dehydrocorticosterone + tetrahydrocorticosterone + 5α-tetrahydrocorticosterone)/(tetrahydrocortisol + 5α-tetrahydrocortisol + tetrahydrocortisone), i.e., corticosterone/cortisol metabolites, and the 17,20-lyase activity by 17-hydroxyprogesterone over androgen metabolites; (17-hydroxy-pregnanolone + pregnanetriol)/ (androsterone + etiocholanolone) and (5-pregnenetriol/DHEA). Delayed diagnosis of 17OHD in one case initially suspected to have primary aldosteronism and a further patient with new onset hypertension and hypokalaemia suggests the likelihood of atypical presentation of rare causes of CAH [58,61].

### 6.1. Lipoid Congenital Adrenal Hyperplasia (LCAH)

LCAH is a rare and severe form of congenital adrenal hyperplasia in which the synthesis of all adrenal and gonadal steroid hormones is impaired by a defect in the conversion of cholesterol to pregnenolone. The defect in LCAH is mainly in the steroidogenic acute regulatory protein (StAR), which promotes the entry of cholesterol into mitochondria, where it becomes the substrate for the cholesterol side-chain cleavage enzyme. The cholesterol cleavage enzyme (P450scc, CYP11A1) is encoded by the *CYP11A1* gene and converts cholesterol into pregnenolone. Although rare, in some patients, P450scc mutations have also been shown to cause LCAH. In the more severe form of LCAH, adrenal glands are enlarged and cholesterol deposits are found. Affected infants die from glucocorticoid and mineralocorticoid deficiency quickly if hormone treatment is not initiated. The absence of pregnenolone production is one of the diagnostic clues, though gene sequencing remains a definitive diagnostic method. Most cases of LCAH caused by severe loss of function mutations present with severe, early-onset adrenal failure and complete phenotypic 46XY sex reversal in genetic males [62,63]. However, patients with late-onset LCAH (non-classic form) due to partial loss of StAR or P450scc activity have been described [64,65]. Using serum samples and LC-MS/MS, two LCAH patients showed a decrease in levels of all steroid hormones as a result of a defect in the StAR protein [66].

### 6.2. Cytochrome P450 Oxidoreductase Deficiency (PORD)

PORD is a rare form of CAH that can present as skeletal malformations, ambiguous genitalia, or menstrual disorders and is caused by cytochrome P450 oxidoreductase mutations, which affect electron transfer to all microsomal cytochrome P450 and some non-P450 enzymes. The latter are non-P450 enzymes involved in cholesterol, sterol, and drug metabolism. POR mutations can disturb steroidogenesis by affecting steroidogenic enzymes such as CYP21A2, CYP17A1 and CYP19A1 (the aromatase). PORD can be divided into classic PORD, which is severe, and non-classic PORD, with less severe symptoms, also termed late or adult-onset PORD. The activities of the three steroidogenic enzymes may be affected to different levels by various mutations, giving rise to various phenotypes [67]. PORD is linked to disorders of sex development as well as skeletal defects. Studies are still required to clarify the relationship between genotype and phenotype. In one study, patients showed decreased or normal baseline DHEA, androstenedione, and DHT, while levels of serum progesterone, pregnenolone, 17OHP, and corticosterone were elevated [68]. Urinary steroid profile in PORD has been measured by GC-MS. Steroid 21-hydoxylase activity was calculated as a ratio of pregnanetriolone to cortisol metabolites (pregnanetriolone)/(tetrahydrocortisone + tetrahydrocortisol + 5α tetrahydrocortisol) and 17 hydroxyprogesterone metabolites/cortisol metabolites (17-hydroxypregnanolone + pregnanetriol)/(tetrahydrocortisone + tetrahydrocortisol + 5α tetrahydrocortisol). 17-hydroxylase activity was defined as the ratio of corticosterone metabolites over cortisol metabolites (tetra-11-dehydrocorticosterone + 5α tetra-11-dehydrocorticosterone + tetrahydrocorticosterone + 5α tetrahydrocorticosterone)/(tetrahydrocortisone + tetrahydrocortisol + 5α tetrahydrocortisol), and 17, 20 lyase activity was defined as the ratio of 17-hydroxyprogesterone metabolites over androgen metabolites (17-hydroxypregnanolone + pregnanetriol/androsterone + etiocholanolone). The ratio of progesterone metabolite pregnanediol over cortisol metabolites was used as a PORD-specific diagnostic ratio. Patient ratios were increased compared to the reference range [69].

## 7. Multiplex Steroid Profiling

The development of steroidomic profiles is helpful for the differential diagnosis of disorders of steroid metabolism and especially for classical and non-classical forms of congenital adrenal hyperplasia. The capability for multiplexed profiling of steroids has been applied to characterise metabolic signatures of changes in steroid metabolism subtypes and is a main advantage of mass spectrometry over the immunoassay. Travers et al. [70] developed an LC–MS/MS method for the simultaneous determination of 15 endogenous corticosteroids in serum. Cortisol, cortisone, 11-deoxycortisol, 17-hydroxyprogesterone, 21-deoxycortisol, progesterone, 11-deoxycorticosterone, corticosterone, 11-dehydrocorticosterone, 18-hydroxycorticosterone, 18-hydroxy-11-deoxycorticosterone, aldosterone, dehydroepiandrosterone sulphate, testosterone and androstenedione were resolved by their method. The authors suggest that in CYP21A2-deficient children, progesterone and 17OHP increased, whereas the metabolites downstream were lower in CYP21A2-deficient patients than in controls. The authors raise the possibility of a differential diagnosis among all subtypes of steroid-related disorders.

In a study of rare causes of CAH and the role of LC-MS/MS in their diagnosis, LC-MS/MS techniques showed an increase in 11-deoxycortisol and 11-deoxycorticosterone levels in the serum of a CYP11B1-deficient patient, a further patient with 3β-HSD deficiency showed a significant increase in DHEA, and a PORD a patient was mainly characterised by elevated 17OHP and progesterone, and impaired synthesis of androgen [66]

The metabolic profiles of serum steroids (primarily cortisol) using LC-MS/MS were studied in CAH subtypes. In this study in a group of 21OHD patients, 17OHP and 21-deoxycortisol were significantly increased, while cortisol and its metabolites were decreased. Higher levels of corticosterone and 18OHcorticosterone were observed in 17OHD, and the ratio of DHEAS/pregnenolone sulphate was decreased in all three patients. A patient with 11β-hydroxylase deficiency demonstrated significantly elevated 11-deoxycortisol and its metabolite tetrahydroxy-11-deoxycortisol, with reduced metabolic ratios of 11β-hydroxytestosterone/testosterone and 11β-hydroxyandrostenedione/androstenedione. [71]

## 8. Conclusions

Steroid profiling can contribute to the diagnosis of abnormalities in steroid metabolism. For each clinical problems, steroid profiles can suggest excess or deficiency. Where a clinical problem is suspected, the data can be more effectively interpreted in its context. Hypertension can be due to one of five rare defects of genes increasing serum aldosterone or primary aldosteronism. Early evidence suggests that steroid profiling can contribute to subtyping the causes of hyperaldosteronism or Cushing’s syndrome [2,3]. Patients with adrenal incidentaloma undergoing examination for signs of adrenal hormone excess can benefit from LC/MS systems that measure steroids and catecholamines in a single analytical run [29]. Recent studies suggest the value of urine steroid metabolomics in the detection of a ‘malignant steroid fingerprint’ in patients with ACC [30]. LC-MS/MS shows a more diagnostic ability for CAH as it can obtain information on a range of steroids and contribute to the significance of the enzyme involved in the pathology of CAH [47].

Modern LC-MS/MS and GC-MS techniques have aided in the quantitative analysis of a large number of steroid metabolites in a single measurement with a higher specificity than found in immunoassays [21]. In most laboratories, steroid profiling with mass spectrometry is not available. It is likely that laboratory centres that meet quality standards will provide essential steroid profiles and have the required skills to provide interpretative reports. Major changes in steroid levels and patterns occur during the neonatal period, adrenarche, puberty, pregnancy, and menopause. Age-related reference ranges of steroid concentrations or ratios are expected when interpreting results.

Mass spectrometry remains a powerful tool by which several steroids can be measured at a single point in time. The alternate pathway is favoured in castration-resistant prostate cancer [72] and in CAH [16]. The interpretation of steroid profile will become more intriguing as our knowledge of steroid biosynthesis and pathology increases with the advent of new technologies.

The integration of mass spectrometry into investigations of steroid pathology remains in its early stages; it plays a more defined role in patients with CAH, which show multiple hormonal imbalances [53]. The European Endocrine Society states that when combined with imaging criteria, urine steroid metabolomics diagnosed adrenocortical carcinoma with higher accuracy than imaging alone [5]. The future lies in a combination of multidisciplinary approaches to the analysis of steroid metabolomics that integrates current investigative techniques, advances in laboratory technology (GC-MS and LC-MS/MS), and progress in genetic analysis.

## Data Availability

No new data were created or analyzed in this study. Data sharing is not applicable to this article.
